# Microfluidic encapsulation of the human gut microbiota—a tool for research and beyond

**DOI:** 10.1038/s41378-026-01264-7

**Published:** 2026-06-23

**Authors:** Sydney K. Wheatley, Lisa Dupeyroux, Melanie Rodger, Hanna Hamoud-Michel, Tommy Boutin, Catherine Prattico, Sophie Lerouge, Corinne F. Maurice, Ali Ahmadi

**Affiliations:** 1 Department of Mechanical Engineering, École de technologie supérieure, Montréal, QC Canada; 2 University of Montréal Hospital Research Center, Montréal, QC Canada; 3 Department of Microbiology & Immunology, McGill University, Montréal, QC Canada; 4 McGill Centre for Microbiome Research, Montréal, QC Canada; 5 Department of Radiology, Radio-oncology, and Nuclear Medicine, University of Montréal, Montréal, QC Canada

**Keywords:** Engineering, Materials science

## Abstract

Over the past few decades, the importance of the human gut microbiota has been cast into the limelight. A growing number of studies are attempting to detangle the complex functions of the gut microbiota for human health; however, one existing shortcoming is an incomplete understanding of the microbiota community composition. Up to 70% of bacteria colonizing the human gastrointestinal tract are estimated to lack complete genomic or functional characterization due to their low abundance within the gastrointestinal tract or challenge to culture. As traditional culture methods often favour fast-growing or easily cultured species, alternative strategies are needed to access the broader gut microbial diversity. Here, we propose a novel approach to improve the growth of difficult-to-culture gut bacteria through single-cell microencapsulation, which will allow for in vitro manipulation. This work provides evidence of high biocompatibility of four-arm poly(ethylene glycol) maleimide (PEG4MAL) for gastrointestinal microbial culture and significant anaerobic gut bacteria proliferation in PEG4MAL microbeads generated via microfluidics. Specifically, we varied the concentration of PEG4MAL and the presence of Arg-Gly-Asp peptide motifs to tune the mechanical properties and porosity of the microbeads, and examined their impact on bacterial viability, confluency, and colony formation.

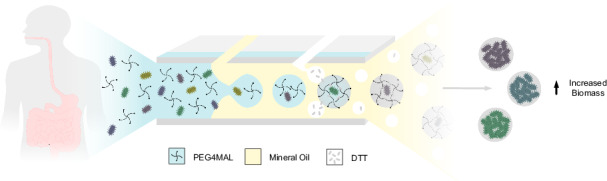

## Introduction

Central to human health, the gut microbiota is made up of a diverse community of bacteria, fungi, archaea, and viruses capable of multidirectional interactions with the human host^1^. One of the most well described roles of the gut microbiota is complex carbohydrate and polysaccharide metabolism, as these compounds are typically nondigestible by host enzymes^2^. In addition, the relative abundance of different members of the microbial community influences host health^3^, as significant shifts in bacterial relative abundance are tightly linked to adverse health outcomes, such as metabolic risk factors, bowel disorders, cancers, and more^4,5^. However, the causal role of the gut microbiota in these conditions remains to be firmly established, as these microbial communities are dynamic and responsive to environmental changes^6^. In addition, the inter-individuality of the gut microbiota makes establishing a causal relationship between the microbiota and human health a daunting task, with many unknowns remaining^7^.

Of the vast number of bacterial taxa capable of colonizing the human gut, it is estimated that up to 71% remain uncultured, which can, in part, be attributed to the complexity in recapitulating the gastrointestinal (GI) niches of growth in vitro^8,9^. These microorganisms, referred to as the “Most Wanted Taxa” of the gut microbiota, are highly sought after for key information such as whole genome data^10^. Existing in vitro systems inadequately mimic essential environmental gut factors, including nutrient availability, pH, degree of oxygenation, interspecies interactions, and mechanical stimuli, limiting our ability to culture these taxa^11^. These issues are often exacerbated for low abundant strains known to be present within the GI tract, posing a significant barrier to advancing human health research and discovery. Successful cultivation would facilitate investigations into microbiota inheritance, function, metabolism, and immune system development, while providing access to high-quality microbial whole-genome data—critical information that remains necessary to our understanding of the gut microbiota’s role in human health ^2,3,11^.

As culture-based methods remain challenging for the gut environment, sequencing-based approaches have dominated gut microbiome research and led to significant data-driven advancements, including the identification of new species, improved diagnostics, and complex analyses^3,12^. Genomic analysis enables rapid and specific detection of bacteria up to strain level using several complementary “omics” approaches; yet these methods fall short with low biomass samples and can be biased by amplification requirements^3,13^. As a result, the independent yet interconnected sequencing- and culture-based approaches would benefit from obtaining whole-cell samples of difficult-to-culture bacteria, invaluable for unbiased genomic analysis and microbiome research.

The human GI tract presents a unique challenge for mimicking culture conditions due to its spatially structured microbial community, pH, enzymatic diversity, and anoxic conditions^14^. Novel culturing approaches, such as in situ culture, allow for the growth of co-dependent bacterial taxa and limit the over-representation of fast-growing species but have not been widely applied to the human gut microbiota^15–17^. Microfluidic technologies have been adapted for microbiome research, enabling the creation and manipulation of microenvironments in a high-throughput fashion^18,19^. For example, the Gut-on-a-chip technology has allowed researchers to recreate aspects of human physiology, incorporating fecal-derived microorganisms and assessing host response^20–22^, with limited exploration of the microbial communities involved^23,24^. Other microfluidic approaches have explicitly focused on the microbial members of the human gut microbiota through the isolation and compartmentalization of bacteria using microarrays or water-in-oil droplets for on-chip culture^18,25,26^. On-chip culture has streamlined antibiotic resistance screening, characterisation of bacteriophage host range, cell-cell interactions, new species detection, and more^27,28^. However, they are limited by insufficient nutrient and waste diffusion, and relatively short experimental times. Therefore, a more robust approach is needed to capitalize on the benefits of microfluidic technologies while better integrating native environmental conditions. While the physical principles underlying droplet generation, such as the influence of viscosity, interfacial tension, and capillary number are well established, their application has not yet been explored for the encapsulation of intestinal bacteria in PEG4MAL functionalized with RGD under anaerobic conditions.

Alternative microfluidic approaches, such as cell encapsulation, have been investigated to assist with such challenges for cell culture. Recently, there has been an interest in exploring mechanostimulatory effects on bacterial growth and fitness^29,30^. The modulation of hydrogel stiffness and porosity for cell encapsulation can offer important insights into microbial enrichment but remains to be thoroughly investigated. Bacteria encapsulation has shown promising results for bioremediation of soil, contaminated water, and wastewater treatment; however, it has yet to be explored in the context of human gut microbiota culture^31,32^. To date, gut bacteria encapsulation has been limited to probiotic delivery, often yielding low cell survival and engraftment due to the harsh encapsulation methods ^33,34^.

Microfluidic encapsulation provides a unique alternative, offering high cell viability, easily tailored microbead sizes, compatibility with many biomaterials, and the ability to achieve high-throughput single-cell encapsulation. Historically, cell encapsulation has been dominated by naturally derived biomaterials such as agarose and alginate; however, bacterial metabolism and the acidic environmental GI conditions can degrade these biomaterials. Synthetic polymers for the encapsulation of gut-derived bacteria could provide a viable alternative. A recent study has focused on evaluating the suitability of poly(ethylene glycol)-based materials for bacteria culture and suggests that maleimide-terminated and thiol-crosslinked poly(ethylene glycol) can support the growth of microbial cell aggregates^35^. Four-armed Poly(ethylene glycol) maleimide (PEG4MAL) is a promising synthetic biomaterial with highly tunable properties and innate biocompatibility. Recent work has utilized PEG4MAL to support the growth of organoids in vivo, enable insulin release from encapsulated pancreatic islets, assist with prevention of knee cartilage degradation, and more^36–38^. However, research on PEG4MAL’s interaction with bacteria remains limited and no studies have explored the encapsulation of anaerobic gut bacteria using PEG4MAL for improved culture. Given its favorable properties—such as tunable porosity, low stiffness, and ease of functionalization—PEG4MAL may provide an optimal microenvironment for supporting the enrichment and growth of gut-derived anaerobic bacteria.

**Fig. 1:**
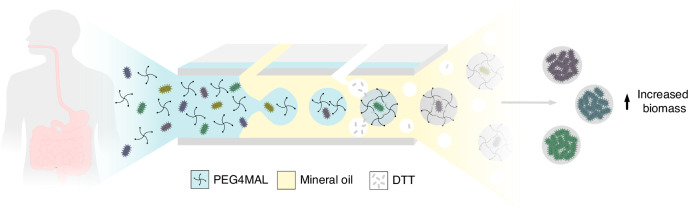
A microfluidic approach to exploring the human gut microbiota through microencapsulation to increase diversity and biomass of culture species

This work introduces a novel application of PEG4MAL to achieve single-cell encapsulation and 3D culture of gut-derived bacteria, providing a platform compatible with the enrichment of previously difficult-to-culture species (Fig. 1). Here, we summarize the characterization of PEG4MAL and the inclusion of RGD peptide to assess compatibility with microfluidic encapsulation of strictly anaerobic GI-associated bacteria. We characterize the effectiveness of microfluidic encapsulation of two representative species of the human gut microbiota, a facultative anaerobe, *Escherichia coli* (*E. coli*), and a strict obligate anaerobe, *Akkermansia muciniphila* (*A. muciniphila*), presenting an effective approach to increase cultured bacterial cell density.

## Experimental methods

### PEG4MAL preparation

The PEG4MAL precursor solution was prepared, as needed, by dissolving 2, 5, 10, and 20% w/v 20 kDa PEG4MAL (Advanced Biochemicals, US) in either HEPES buffer (Thermofisher Scientific, CA), or a mix of HEPES and phenol red (Millipore Sigma, CA) at a 1:1 ratio^37,38^ for microfluidic experiments. A 20 mM crosslinking solution of Dithiothreitol (DTT) (Millipore-Sigma, US) was prepared in phosphate-buffered saline (PBS) (VWR, CA), and all solutions were 0.22 µm filter sterilized (Millipore Sigma, CA).

### Microfluidic optimization

**Fig. 2:**
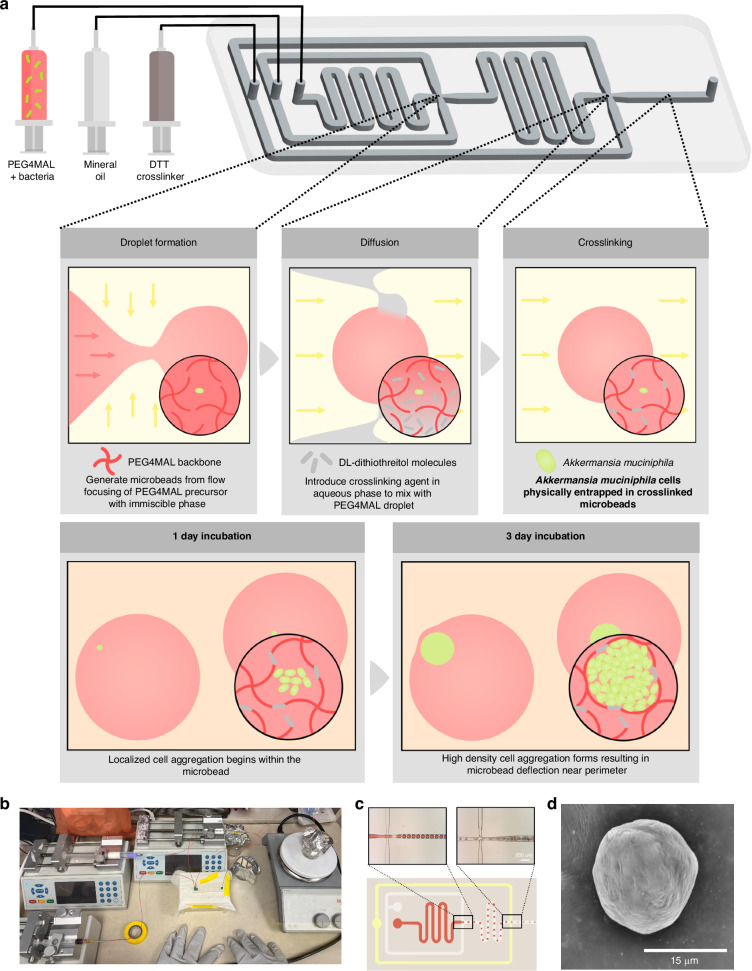
The setup for microfluidic encapsulation including. **a** a schematic of the dual-junction microfluidic chip, droplet formation and crosslinking, and cell growth within microbeads; **b** the microfluidic setup in an anaerobic chamber; **c** microscopy images showing droplet formation at the first junction and microbead merging with DTT at the second junction in the microfluidic chip; **d** a SEM image of a 5% w/v PEG4MAL microbead

A double-emulsion microfluidic chip with 140 µm channel diameter (Microfluidic ChipShop, DE) was used to generate water-in-oil microbeads, with flow controlled by three syringe pumps (Chemyx, US) (Fig. 2a, d). Flow rates were optimized experimentally by ranging the oil-based continuous phase from 0.001 µL/min to 5 µL/min to confirm droplet formation within the dripping regime visually. The continuous phase consisted of mineral oil (Millipore Sigma, CA) and 3% v/v SPAN 80 surfactant (Millipore Sigma, CA)^31^. The PEG4MAL precursor and crosslinker channel flow rates were constant at 0.50 µL/min and 2.85 µL/min, respectively. The range of flow rates was determined based on previously published work^37^. All tubing and connections were sterilized via autoclaving, and the microfluidic chip was sterilized using cyclic passing of 70% ethanol and sterile de-ionized water. Following microbead generation, the collected microbeads were washed to remove the oil-based continuous phase. For washing, the microbead suspension was sequentially filtered through a 200 µm cell strainer, followed by a 70 µm cell strainer (PluriSelect, US). Microbeads retained on the 70 µm cell strainer underwent repetitive washing with media and filtration to remove residual oil ^31^.

To assess the parameters influencing microfluidic droplet generation, surface tension and viscosity were measured in triplicate. Interfacial tension was characterized using a DSA30R tensiometer (Krüss Scientific, DE) and performed in triplicate over 60 seconds. The pendant drop method and manual depositor were used to form a suspended droplet of the PEG4MAL precursor in mineral oil containing surfactant to assess the interfacial tension between the two immiscible fluids. The viscosity of the PEG4MAL precursor solutions and mineral oil with surfactant was assessed using a Physica MCR 301 Rheometer (Anton Paar, DE). Experimental conditions included a parallel plate geometry with a 1 mm gap sealed with oil, and a frequency shear sweep was conducted from 100 s^−1^ to 1000 s^−1^ at 22 °C. Lastly, the osmolality of the PEG4MAL precursor solutions was measured in triplicate using a Model 3320 Osmometer (Advanced Instruments Inc., US) calibrated with a 290 mOsm/L solution. The osmolarity (mOsm/L) was then calculated by multiplying the measured osmolality (mOsm/kg) by the solution density, assuming ideal solution behavior, to ensure isotonic conditions for bacterial encapsulation.

Following assessment of fluid properties, the capillary number,

1 $$\mathrm{Ca}=\,\frac{\mu U}{\sigma },$$

was calculated assuming a shear rate-independent dynamic viscosity to determine the different flow regimes of droplet generation where *U* represents fluid velocity (m/s), *µ* represents dynamic viscosity (Pa·s), and *σ* represents interfacial tension (mN/m)^39^. Microbeads assessed for size were separated from the oil phase by the addition of PBS but were not subjected to filtering. The microbeads were images using an inverted brightfield Revolve Microscope (Echo, US).

### Biomaterial characterization

The mechanical properties of PEG4MAL microbeads were assessed using a MicroTester (CellScale, CA). Washed microbeads were resuspended in PBS and pipetted onto the MicroTester sample stage for isolation and compression. A 76.2 μm diameter beam and 2 mm by 2 mm plate were used to compress the 2% and 5% PEG4MAL microbeads, and a 203.2 μm diameter beam was used for 10% and 20% PEG4MAL microbeads. The Young’s modulus was calculated by fitting the force–deformation data obtained from compression tests to the linear region of the stress–strain curve up to 20% strain. The microstructure was assessed visually using scanning electron microscopy of freeze-dried samples. To prepare the samples for imaging, 20 μL droplets were formed by manually adding PEG4MAL to the crosslinker at a ratio of 4:1, which underwent immediate crosslinking^36^. The droplets were freeze-dried for 8 hours (HarvestRight, US) and sputter-coated with a 20 nm layer of gold nanoparticles using a K550x Sputter Coater (Quorum, UK). To assess swelling, crosslinked droplets of PEG4MAL were prepared, as previously described, and submerged in de-ionized water. After 24 hours, the samples were freeze-dried, as previously described. The mass swelling ratio^40^

2 $${Q}_{m}=\frac{{m}_{\mathrm{swollen}}}{{m}_{\mathrm{dry}}},$$

was determined as a function of the swollen mass (${m}_{{\rm{swollen}}}$) to the freeze-dried mass (m_dry_)^40^. Due to the rapid thiol-based reaction between PEG4MAL and DTT, heterogeneous crosslinking can occur at high PEG4MAL volumes; therefore, the maximum droplet volume investigated was 20 μL for both microstructure analysis and swelling, and tests were otherwise conducted with microbeads ^41^.

### Effect of PEG4MAL on bacterial growth

To verify the biocompatibility of PEG4MAL, a resuspended culture of bacteria was exposed to crosslinked droplets of PEG4MAL in triplicate, produced as previously described. *E. coli* was used as a model species to ensure rapid cell proliferation, enabling quick assessment of the response to PEG4MAL exposure. *E. coli* was grown overnight from frozen stock in Brain Heart Infusion (BHI) media under aerobic conditions at 37 °C. The optical density was recorded using a spectrophotometer (Thermofisher Scientific, CA) and diluted to reach a final concentration of approximately 9 × 10^7^ cells. mL^−1^. A 3 mL aliquot of the diluted suspension was added to a culture tube containing a 40 μL droplet of crosslinked PEG4MAL. On days 1, 2, and 3, aliquots were incubated with AlamarBlue (Thermofisher Scientific, CA) for 4 hours in the dark to evaluate cell metabolic activity. Post-incubation, an aliquot was pelleted at 9500 × *g* for 5 minutes, and the supernatant was removed for optical density measurements of the sample (*A*) and the control of media and AlamarBlue (*C*). The percent reduction of AlamarBlue,

3 $$\mathrm{Reduction}\left( \% \right)=\frac{{E}_{{600}_{\mathrm{oxi}}}{A}_{570}-{E}_{{570}_{\mathrm{oxi}}}{A}_{600}}{{E}_{{570}_{\mathrm{red}}}{C}_{600}-{{E}_{600}}_{\mathrm{red}}{C}_{570}},$$

was calculated based on absorbance readings at 570 nm and 600 nm using manufacturer values for molar extinction coefficients (*E*). In addition, the culturability of cells post-exposure to crosslinked PEG4MAL droplets was measured by serially diluting and plating aliquots of each condition on BHI agar using the spot titer method. The culture plates were incubated aerobically at 37 °C overnight and imaged for colony counting.

### Bacteria microencapsulation

The optimal concentration of PEG4MAL for bacterial microencapsulation was determined by observing cell survival and growth following encapsulation. To achieve single-cell encapsulation the required cell concentration for suspension in the PEG4MAL precursor was calculated based on the size of the microbeads, assuming a Poisson distribution. *E. coli* was grown overnight as previously described, and the diluted culture was resuspended in 1 mL of each concentration of the PEG4MAL precursor solutions. Microbeads were generated by microfluidic system and washed, as previously described. The encapsuled *E. coli* cells were resuspended in BHI and grown under aerobic conditions at 37 °C. After 24 h, the microbeads were stained using 0.0015% nucleic acid dye SYTO 9 and imaged with an Axiovert 3 epifluorescence microscope (Zeiss, DE). Z-stacking was performed for a 70 μm region with 0.2 μm slice thickness to visualize 3D colony structure within the microbead.

Cell viability was obtained by staining encapsulated cells with the Live Dead Assay of Propidium Iodide (PI) and SYTO 9 fluorescent stains (Thermofisher Scientific, CA), per the manufacturer’s instructions. Samples were imaged using an Axiovert 3 microscope and images were assessed by fluorescent area coverage. Based on these results, subsequent experiments involved the encapsulation of bacteria in 5% w/v PEG4MAL via microfluidics, as previously described, using a 2 μL/min, 0.5 μL/min, and 2.85 μL/min flow rate for the oil, PEG4MAL, and crosslinker phase, respectively.

All experiments were then repeated under anaerobic conditions (Coy lab products, USA) by maintaining a controlled environment atmosphere of 5% H₂, 10% CO₂, and 85% N₂ for *A. muciniphila*, a strict anaerobe (Fig. 2b). The encapsulated cells were stained and imaged as previously described. A 2D analysis of the colony formation was assessed by comparing the size of cell aggregation within the microbead to that of resuspended cell culture.

The addition of plasticizers was explored to investigate the impact of mechanical properties of microbeads on cell activity. Arginylglycylaspartic acid (RGD peptide) was selected to act as a plasticizing agent as it is a small organic molecule that does not chemically interact with the crosslinking agent or the PEG4MAL backbone. In mammalian cell culture, RGD binds to the cell membrane; however, the same binding motifs are not present on microbial membranes. The inclusion of 9 μM RGD in the PEG4MAL precursor solution was hypothesized to reduce the crosslinking efficiency via steric hindrance. The fluid and mechanical properties, as well as response of encapsulated cells were characterized for the RGD-containing PEG4MAL biomaterial, as previously described.

### Statistical analysis

Results are presented as mean ± standard deviation for normally distributed data or otherwise as median, interquartile range, or as a percentage of population. Statistical analyses were performed in RStudio and a *p* value < 0.05 was considered significant. The Shapiro-Wilk test was used to test for normality and skewness of the data and the Levene’s test was used to assess the equality of variances. For normally distributed data, a one-way ANOVA was performed with a Tukey’s post-hoc analysis.

## Results & discussion

### Microfluidic optimization of PEG4MAL microbead generation

The dual-junction microfluidic chip enabled the controlled formation of microdroplets (Fig. 2c). The coefficient of diffusion for DTT in PBS was reported as 2.83 × 10^−6^ *cm*^2^/*s*, thus enabling rapid downstream crosslinking prior to collection^42^. The setup was successfully integrated into an anaerobic chamber, ensuring encapsulation under conditions conducive to survival of anoxic bacteria (Fig. 2b). Herein, this section discusses the assessment of fluid properties and droplet generation behaviour within the microfluidic chip.

**Fig. 3:**
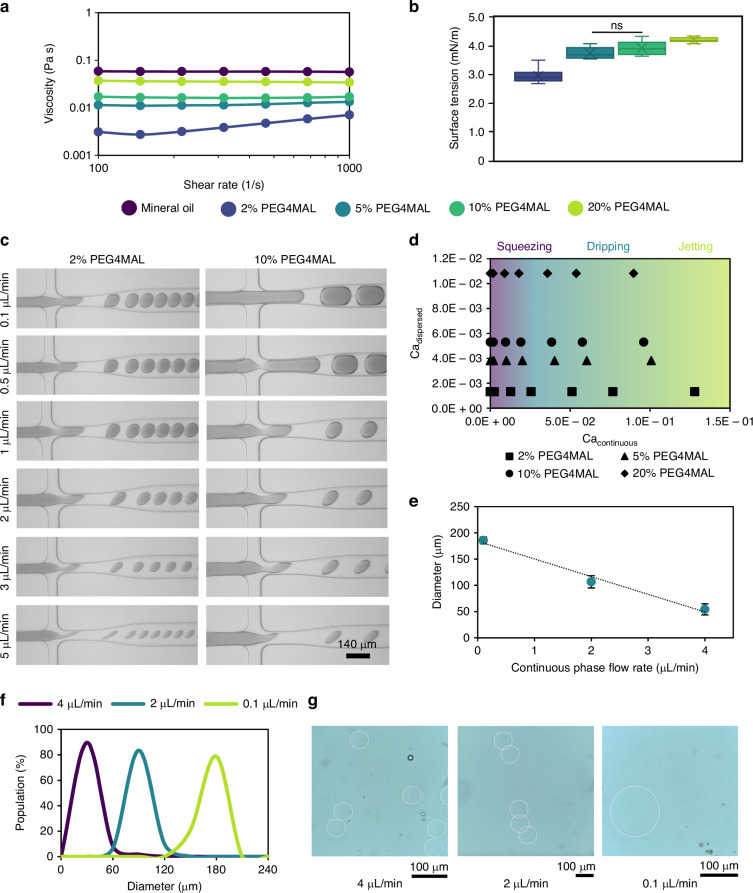
Fluid properties of PEG4MAL and characterization of the microfluidic encapsulation including. **a** The viscosity of various PEG4MAL concentrations and mineral oil with surfactant, *n* = 3; **b** the interfacial tension between various PEG4MAL concentrations and mineral oil with surfactant, *n* = 39; **c** representative captures of PEG4MAL droplet generation with varying continuous phase flow rates, while the dispersed phase was kept constant at 0.5 µL/min; **d** the capillary number range for the continuous and dispersed phases for the different PEG4MAL concentrations; **e** trend of final microbead size by varying the continuous phase flow rate (*n* = 500), with dispersed phase flow rate of 0.5 µL/min and crosslinker flow rate of 2.85 µL/min; **f** a histogram of microdroplet size distributions; **g** representative optical microscopy images at different continuous phase flow rates (*n*= 500)

The viscosity of PEG4MAL at concentrations of 2, 5, 10, and 20% w/v was constant within the observed shear rate range and measured as 4.4 × 10^−3^, 1.2 × 10^−2^, 1.7 × 10^−2^, and 3.6 × 10^−2^ Pa·s, respectively, with viscosity trending upwards with increasing PEG4MAL concentration (Fig. 3a). The viscosity of the mineral oil containing surfactant was also constant and determined to be 5.8 × 10^−2^ Pa·s (Fig. 3a). All fluids displayed Newtonian characteristics as there were no shear-rate dependencies observed over the same shear rate range previously reported in literature^43^. The minimal apparent variations in the 2% w/v PEG4MAL viscosity were assumed to be a result of evaporation and plate slipping. At higher concentrations of PEG4MAL, the increased number of chains per volume contributes to a resistance to fluid movement, thus corresponding to the rise in observed viscosity. Lower viscosity ratio is advantageous for controlled droplet generation within the dripping regime ^39^.

Another important fluid property influencing droplet formation is the interfacial tension between the continuous and dispersed phases. The interfacial tension of PEG4MAL precursor droplets suspended in oil with surfactant was measured as 2.95, 3.75, 3.93, and 4.21 mN/m for 2, 5, 10, and 20% w/v PEG4MAL, respectively with interfacial tension increasing with concentration (Fig. 3b). The increase in interfacial tension at higher PEG4MAL concentrations can be attributed to the corresponding increase in chain density of the precursor solutions and as a result higher interfacial energy. For microfluidic droplet generation, solutions with lower surface tension, such as 2% and 5% w/v PEG4MAL, were more desirable for better control over droplet size.

Collectively, fluid viscosity and interfacial tension data were used to calculate the capillary number over a range of fluid velocities (Fig. 3c). The capillary number is a key dimensionless parameter that determines the dominant force driving droplet generation, with lower values indicating a greater influence from surface tension. Combined with visual observation, fluid regimes—squeezing, dripping, and jetting—were classified. For the squeezing behaviour, the droplets appear elongated as they are larger than the channel diameter. Better control of the droplet size is achieved in the dropping behaviour as the droplets form at the junction into stable droplets smaller than the channel. Lastly, jetting is described as the chaotic breakoff of droplets downstream of the junction from a thin jet of the dispersed phase. Experimentally, the flow rate of the continuous phase was varied to visually confirm the transitions between these regimes, which can be associated with capillary number thresholds, while maintaining a constant flow rate for the discrete phase (Fig. 3d). Critical capillary numbers for transition states are dependent on channel geometry, the ratio of fluid viscosities, and are influenced by user interpretation; however, the observed transitions were within a similar range to reported values of squeezing to dripping and dripping to jetting occurring at continuous phase capillary numbers approaching 10^−2^ and 10^−1^, respectively^39,40^. By manipulating the continuous phase flow rate, microbead size could be modulated within a narrow range. For example, by increasing the flow rate of the continuous phase, the size of the microbeads decreased, and this precise control is particularly advantageous for achieving single-cell encapsulation (Fig. 3e, f). Maintaining operation within the dripping regime was prioritized to achieve controlled and uniform microbead production, leading to the selection of an optimal continuous phase flow rate of 2 μL/min. However, further optimization of the crosslinker flow rate is required to further reduce microbead size discrepancies. Overall, a microfluidic approach was successfully integrated to form PEG4MAL microbeads at various concentrations with optimal microbead size ranges.

### PEG4MAL provides an adequate microenvironment for bacterial enrichment

**Fig. 4:**
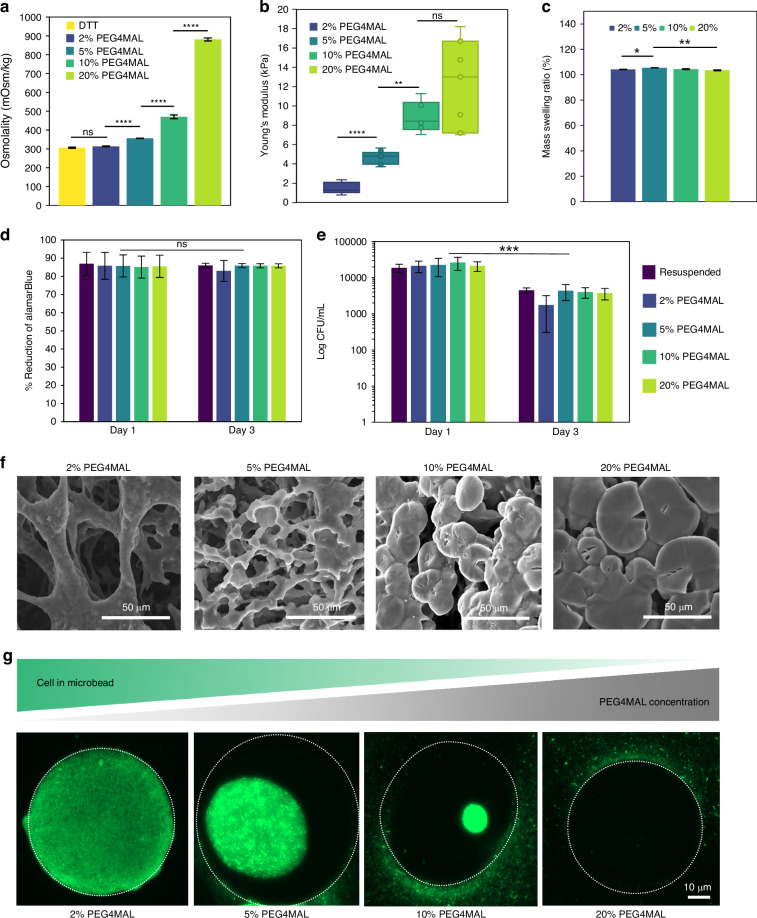
The fluid, mechanical and biocompatibility properties of of PEG4MAL as defined by. **a** The osmolality of various PEG4MAL solutions prior to crosslinking, *n* = 3, *p* < 0.0001; **b** the Young’s modulus calculated at 20% strain, *n* ≥ 6, and *p* < 0.00001; **c** the mass swelling ratio of 20 µL PEG4MAL crosslinked droplet, *n* = 5, *p* < 0.001; **d** the metabolic activity measured by AlamarBlue reagent reduction, *n* = 3, *p* = 0.973; **e** the culturability measured by CFU/mL, *n* = 4 and *p* < 0.0001; **f** the microstructure of PEG4MAL by SEM after freeze-drying; **g** the behaviour of E. coli encapsulated in varying PEG4MAL concentrations after a one-day incubation

The osmolality of PEG4MAL was assessed and determined to follow the same increasing trend with concentration (Fig. 4a). Osmolality is known to play a crucial role in bacteria’s survival, morphology, and function^44^. Some species exhibit osmotolerance or osmoadaptation, where they express internal mechanisms to regulate the response to changes in osmotic pressure^44^. Due to this inherent plasticity, bacteria are capable of surviving in a wide range of environmental osmolalities. Within the human body, for example, the microbiota exists within a physiological range similar to that of mammalian cell culture, which is typically between 260 and 320 mOsm/kg; however, there is a greater variance within gastrointestinal tract depending on a fed or fasted state^45^. As a result, bacteria sampled from the gastrointestinal tract would experience an osmotic shock when suspended in 10% (471 mOsm/L) and more importantly in 20% w/v PEG4MAL precursor solutions (881 mOsm/L). Lower concentrations of PEG4MAL were therefore favoured to prevent hyperosmotic conditions for the cells; however, osmotic perturbation may be a promising avenue to explore novel metabolite production.

As expected, adjusting PEG4MAL concentration also enabled tuning of the mechanical properties. The Young’s modulus was measured to range from 1.5 to 12 kPa for 2% to 20% w/v PEG4MAL as determined via individual microbead compression, with stiffness increasing with concentration (Fig. 4b). There was a larger recorded variance for 20% PEG4MAL microbeads, which may be a result of a heterogeneous microstructure due to rapid crosslinking. Additionally, a larger microbeam was required to compress microbeads formed by higher concentrations of PEG4MAL, which could account for the increase in measurement variability. At low concentrations of PEG4MAL, the magnitude of the Young’s modulus approached that of the ileum mucosal layer, a primary region for gut microbiota growth, which was sought-after for in vitro gut assimilation ^46^.

Additionally, some slight differences in swelling between the PEG4MAL concentrations were observed after 24 hours of immersion in water (Fig. 4c). The significant decrease in swelling ratio observed at higher concentrations of PEG4MAL is assumed to be a result of the denser network structure and potentially a higher degree of crosslinking. Lastly, the ratio of PEG4MAL to crosslinking agent has also been shown to directly impact network structure and resulting swelling capabilities of the hydrogel^38^. Swelling of the biomaterial is important to help adjust for the internal volume increase from the microbial cell proliferation over time.

PEG4MAL has previously demonstrated a high degree of biocompatibility for mammalian cell culture, but evidence supporting its biocompatibility for bacterial culture remains limited^35^. *E. coli* exposed to crosslinked PEG4MAL maintained high metabolic activity—evidenced by 85.87% reduction (±1.39) of the AlamarBlue signal during exposure to 5% w/v PEG4MAL—even after three days in culture, showing no significant difference from standard culture and with low variability (Fig. 4d). The high metabolic activity confirms that the presence of PEG4MAL does not negatively affect cell function. When looking at culturability, there was a significant decrease in colony forming units from day 1 to day 3 for all conditions, including the resuspended control (Fig. 4e). As such, it can be concluded that microbial exposure to PEG4MAL does not elicit a shift to a sporulative or viable but non-culturable (VBNC) state non-conducive to cell culture. Although this study focused on short-term viability, PEG4MAL microcapsules are compatible with gentle enzymatic or chemical degradation, allowing retrieval of viable bacteria for downstream applications, suggesting feasibility for long-term culture and functional assays.

### Tunable properties of PEG4MAL can modify bacteria behaviour

Variations in PEG4MAL microstructure were apparent across the different concentrations observed, with a denser network observed at higher concentrations (Fig. 4f). The consequences of encapsulation on bacterial growth characteristics within these crosslinked networks were investigated. Fluorescently stained *E. coli* enabled visualization of cell distribution across different PEG4MAL concentrations (Fig. 4g). Notably, after 24 h in 2% w/v PEG4MAL, cells were evenly distributed through the microbead in a two-dimensional (2D) projection. In contrast, at 20% w/v PEG4MAL, a greater degree of cell escape and preferential adhesion to the microbead surface was observed. Lower PEG4MAL concentrations could also increase cell escape from the beads and migration to neighboring microbeads. Although some migration between beads can occur, careful selection of PEG4MAL concentration and bead size minimizes cross-contamination, preserving the isolation of individual bacteria and enabling reliable enrichment of less active or low-abundance species. This behaviour aligns with established knowledge of bacteria biofilm formation, wherein bacteria preferentially adhere to stiffer surfaces^30^. Additionally, with a greater degree of porosity at 2 and 5% w/v PEG4MAL, lower PEG4MAL concentrations were favoured to enhance cellular retention and fitness within the microbead.

It has been shown that external mechanical forces acting on cells, such as those experienced within a biofilm, can dictate bacterial orientation during cell division and correspondingly influence cell interactions^47^. As such, there has been a recent interest in exploring the mechanostimulatory effects on bacterial growth and the modulation of hydrogel stiffness for in vitro cell encapsulation can offer important insights^29^. In addition to the physical environment, the presence of solute is also important for bacteria. It has been hypothesized that biofilms also react to extreme osmotic conditions by expanding under hyperosmotic stress and shrinking during hypoosmotic conditions^29^. As a result, the behaviour of biofilms can promote microbial survival by controlling the diffusion of solutes. Studies also suggest that osmotic pressure differences between the biofilm and the surrounding environment can act as a driver for colony growth^48^. Therefore, osmotic pressure differences in between the PEG4MAL microbead and its environment may support cellular proliferation, particularly under conditions of greater porosity and reduced polymer density, which allow for finer control over the internal environment.

A significant degree of 2% PEG4MAL bead loss occurred during cleaning as microbeads were retained on the filters used to remove residual oil, decreasing overall throughput. Therefore, subsequent experiments used 5% w/v PEG4MAL as a balance between throughput and microbial response; however, future investigation into additional PEG4MAL concentrations is recommended.

### PEG4MAL microencapsulation enables high density isolated colony formation of aerobic and anaerobic gut bacteria

**Fig. 5:**
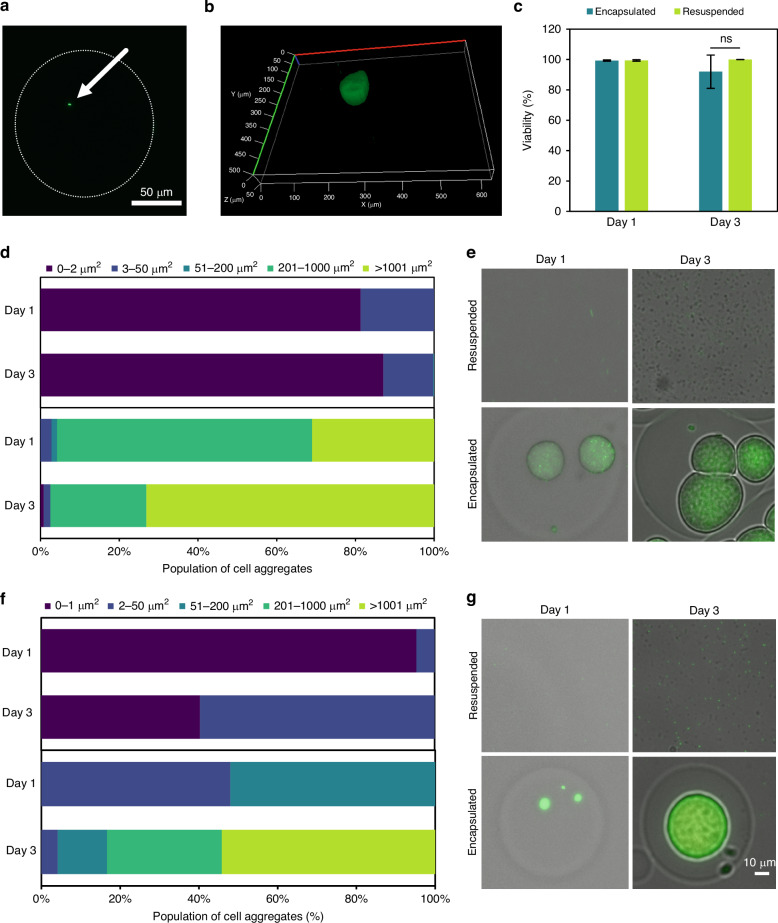
The colony formation in PEG4MAL microbeads characterized by **a** a 5% PEG4MAL microbead containing a single cell of *E. coli* at day 0 prior to incubation; **b** z-stacking of *A. muciniphila* encapsulated in 5% PEG4MAL after a 3-day incubation at 0.1 µm slice thickness; **c** the viability of *A. muciniphila* encapsulated in 5% PEG4MAL microbeads, *n* = 5, *p* = 0.55; **d** the area of *E. coli* aggregation in microbeads vs. resuspended cells, *n* ≥ 71, *p* < 0.0001; **e** encapsulated and resuspended *E. coli* after a 1 and 3 day incubation; **f** the area of *A. muciniphila* aggregation in microbeads vs. resuspended cells, *n* ≥ 24, *p* < 0.0001; **g** encapsulated and resuspended *A. muciniphila* after a 1 and 3 day incubation

The role of single-cell encapsulation is important for future GI-derived community cultures to spatially isolate individual cells, which may lead to improved growth and ease of sorting. Through microfluidic encapsulation and subsequent incubation, it was observed that single-cell-laden microbeads led to the formation of an individual colony/cell aggregation under both aerobic and anaerobic conditions (Fig. 5a, b). *A. muciniphila* maintained high viability with around 90% viability observed after 3 days in culture (Fig. 5c). No indication of a necrotic core within the microbead was found, indicating sufficient diffusion of nutrients and waste. To quantify the abundance of cells, a 2D visual analysis using image thresholding (ImageJ) enabled a comparison between cellular aggregation within the microbead to that of a resuspended culture by measuring coverage area^35^. A significant increase in bacterial aggregation was observed for encapsulated *E. coli* compared to resuspended culture (Fig. 5d, e). Additionally, the same significant increase was observed for encapsulated *A. muciniphila* incubated under anaerobic conditions (Fig. 5f, g). Although the doubling time for *A. muciniphila* is significantly less than *E. coli*, the rapid proliferation of encapsulated *A. muciniphila* suggests preferential growth within a substrate as opposed to in suspension. In the GI tract, *A. muciniphila* primarily resides within the mucosal layer and is well-characterized for its mucin-degrading properties^49^. It is hypothesized that matrix encapsulation contributed to an altered phenotype for rapid cell division, which may be a promising approach to culture mucosal layer-derived gut bacteria in vitro. The high-density growth of difficult-to-culture cells within the microbead facilitates the collection of higher biomass samples, thereby enabling rapid sorting and reduced analytical bias.

### PEG4MAL manipulation with RGD modifies mechanical properties and cellular response

**Fig. 6:**
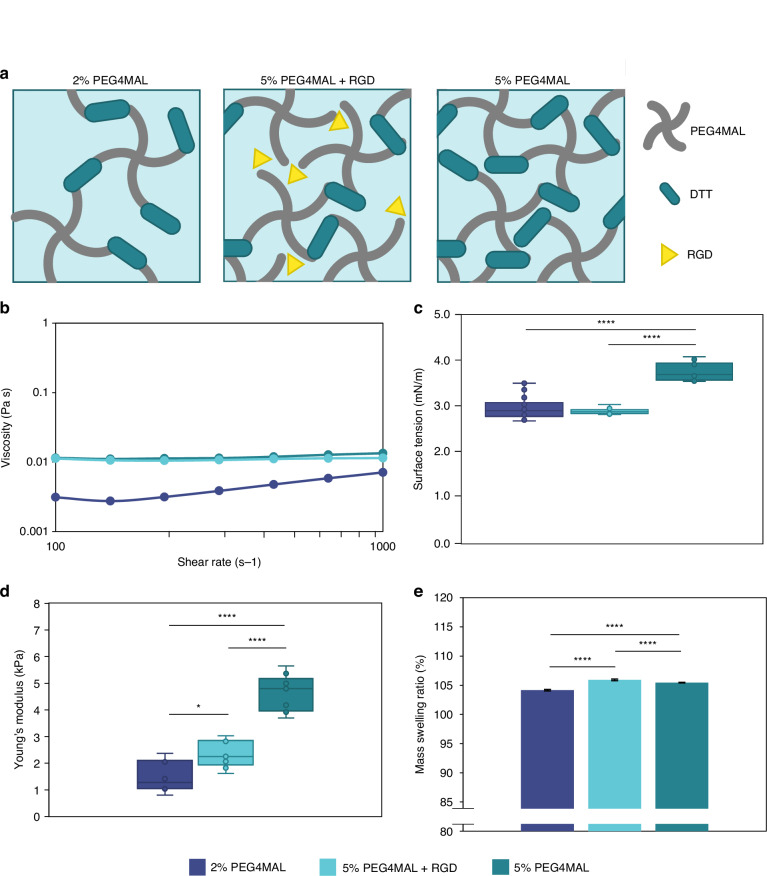
The fluid properties of PEG4MAL with RGD including. **a** an illustration of 2% PEG4MAL, 5% PEG4MAL, and 5% PEG4MAL with RGD precursor solutions; **b** the viscosity of the precursor solutions *n* ≥ 3; **c** the interfacial tension of the precursor solutions; **d** the Young’s modulus calculated at 20% strain, *n* ≥ 8; **e** the mass swelling ratio, *n* = 5

**Fig. 7:**
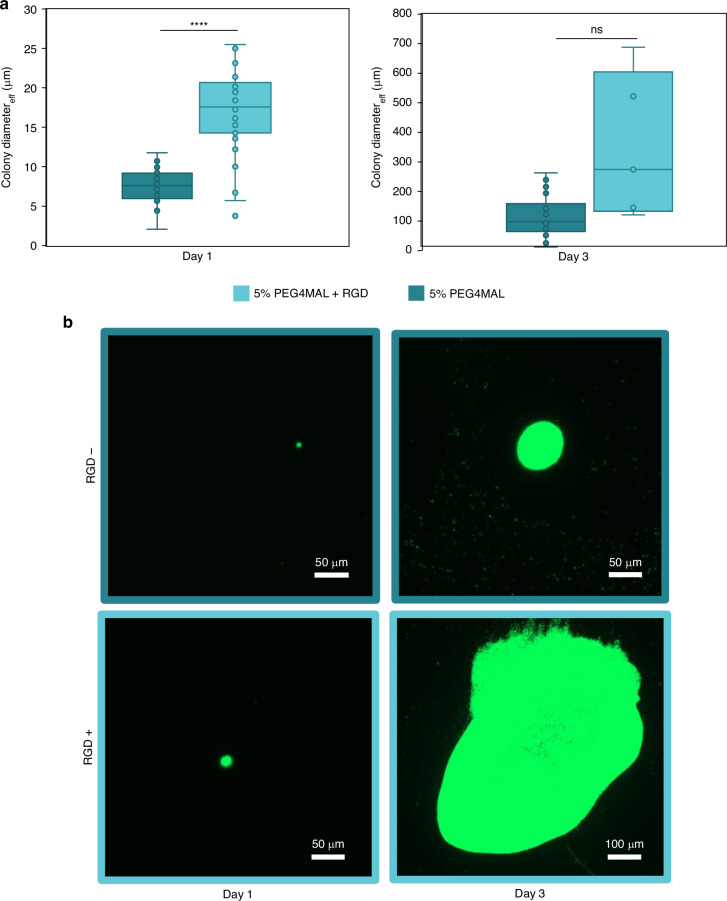
The cellular response to encapsulation in PEG4MAL with RGD. **a** Assessment of the effective colony diameter of encapsulated cells on day 1, n ≥ 5 and assessment of the effective colony diameter of encapsulated cells on day 3, n ≥ 5; **b** representative images of colony-containing microbeads at day 1 and 3 of incubation

RGD is a bioactive peptide motif found within the mammalian extracellular matrix and is widely studied in PEG4MAL-based encapsulation systems for its ability to mediate mammalian cell adhesion^40^. While its use in microbial encapsulation remains limited, RGD was selected as a proof-of-concept compound to explore the impact of a peptide-based plasticizer to reduce crosslinking and mechanical properties (Fig. 6a). Additionally, the constituent amino acids of the RGD sequence may be utilized by encapsulated bacteria to support metabolic or cellular processes. As previously mentioned, low concentrations of PEG4MAL, namely 2%, offered enhanced cellular response to encapsulation; however, practical limitations impeded its application. The inclusion of the bioactive RGD peptide into the PEG4MAL precursor was assessed to understand its impact on modifying the mechanical properties of PEG4MAL. No differences were observed in the viscosity of RGD-containing 5% w/v PEG4MAL compared to without (Fig. 6b). Regarding the interfacial tension, there were significant differences observed as RGD acted as a surfactant to reduce the interfacial tension to a magnitude similar to 2% w/v PEG4MAL (Fig. 6c). The lower interfacial tension of the 5% w/v PEG4MAL with RGD results in higher throughput droplet generation. The addition of RGD decreased the stiffness of the crosslinked 5% w/v PEG4MAL network and significantly increased swelling capacity (Fig. 6d, e). It is presumed that the RGD peptide reduces the crosslinking efficiency, thus producing a greater degree of porosity and a less dense microstructure. Lastly, due to the greater intra-microstructure space available for cell growth, a significant increase in *A. muciniphila* aggregation was observed compared to 5% w/v PEG4MAL (Fig. 7a, b). These effects are primarily physical, modulating network stiffness and porosity, rather than providing biochemical cues that directly influence bacterial metabolism.

Exploring the use of other plasticizing agents that interact with encapsulated cells for site adhesion, such as mucins, is a promising future avenue to better characterize the importance of physical cues for gut microbiota in vitro enrichment. This study demonstrates that the mechanical characteristics of the encapsulating matrix go beyond structural support by actively shaping microbial fitness and behaviour thus revealing biomechanical forces as a key regulator of microbial enrichment.

## Conclusion

This work demonstrates that PEG4MAL is an adequate biomaterial for gut bacteria encapsulation due to its high degree of biocompatibility, resistance to degradation from harsh pH conditions, and tunable porosity. We also determined that the concentration of PEG4MAL influenced encapsulated cell behaviour, with low concentrations of PEG4MAL (<5%) more desirable for bacteria confluency within the microbead. Further enhancement of microbial proliferation within the microbead could be achieved by modifying the PEG4MAL structure using RGD. Over a short period of time, significant colony formation within the microbead was observed compared to standard resuspended culture, which translates to sufficient sequencing biomass using only 1–2 microbeads. Physical cues from the surrounding PEG4MAL network were shown to impact the proliferation of cells within the microbead. However, this work does not provide a comprehensive assessment of the impact of encapsulation for all dominant phyla present within the GI tract. Additionally, a better characterization of PEG4MAL concentrations between 2% and 5% remains warranted, as well as the use of other small molecules, other than RGD, that may induce desirable changes in mechanical, fluid, and adhesion properties of PEG4MAL.

Future work is recommended to better characterize the importance of mechanostimulation of the gut microbiota during in vitro culture. Understanding the role of peristaltic forces within the GI tract and mechanical properties of the mucosal layer may be key to unlocking new in vitro experimental models. This work provides foundational evidence of the benefits of GI microbiota encapsulation; however, future investigation is required to examine the impact of community-based encapsulation and co-culture. By leveraging microfluidic encapsulation of the gut microbiota, future community- and co-culture-based studies may provide new insights into the diverse microbial interactions within the GI tract as well as the production of novel bioactive compounds important for human health. This approach is well-suited for integration with in situ culture, whereby encapsulated bacteria are passaged again in their natural environment, to allow access to nutrients and chemical signaling challenging to recreate in vitro. Collectively, this work will provide researchers with the tools to change the uncultured majority into a minority in the gut and overcome current limitations in our understanding of microbial interactions.

## Supplementary information

41378_2026_1264_MOESM1_ESM

41378_2026_1264_MOESM2_ESM
