# Supplementary material for: Microfluidic encapsulation of the human gut microbiota—a tool for research and beyond
Source: Microsyst Nanoeng. 2026 Jun 23;12:245. doi: 10.1038/s41378-026-01264-7 (PMC13287474; doi:10.1038/s41378-026-01264-7)
Supplement: Supplementary file 2 — 41378_2026_1264_MOESM2_ESM [file 41378_2026_1264_MOESM2_ESM.pdf]

Microfluidic Encapsulation of the Human Gut Microbiota – A Tool for Research and Beyond

Sydney K. Wheatley[1,2], Lisa Dupeyroux[1,2], Melanie Rodger[1,2], Hanna Hamoud-Michel[1,2], Tommy Boutin[4], Catherine Prattico[4], Sophie Lerouge[1,2,3], Corinne F. Maurice[4]*, Ali Ahmadi[1,2]*

[1]École de technologie supérieure, Montréal, Canada [2]University of Montréal Hospital Research Center, Montréal, Canada [3]University of Montréal, Montréal, Canada [4]McGill University, Montréal, Canada

*Corresponding authors: ali.ahmadi@etsmtl.ca; corinne.maurice@mcgill.ca

Supplementary Materials

To assess the stability of the PEG4MAL microstructure, crosslinked droplets were manually formed by adding 16 µL of the PEG4MAL precursor to 4 µL of DTT. Simulated gastric and intestinal fluids were prepared by using hydrogen chloride (VWR, CA) and sodium hydroxide (VWR, CA) to recapitulate the range of pH observed within the gastrointestinal tract [S1]. Crosslinked droplets were submerged in pH 1, 6, or 7.5 solutions for 4, 24, or 6 hours, respectively. An additional set of crosslinked droplets were subjected to sequential exposure to each condition. Crosslinked droplets were freeze-dried (HarvestRight, US) for 8 hours. Prior to imaging, the samples were sputter coated with a 20 nm layer of gold nanoparticles using a K550X Sputter Coater (Quorum, UK) and imaged with a TM3000 scanning electron microscope at 15kV (Hitachi, US). Degradation was assessed visually by measuring the void fraction (Ø) of 2D binary images for all conditions (Fig. S1).

Additionally, the hard-to-culture Gram-positive bacterium *Clostridium beijerinckii* (*C. beijerinckii*) was cultured using the same method previously applied for *A. muciniphila*. Observations were performed by fluorescence microscopy at 20x magnification using an ECHO Revolve microscope (Discover Echo, US) (Fig. S2). As shown in Fig. S2, imaging at day 1 and day 3 revealed the progressive formation of discrete bacterial colonies within the microbeads. Notably, such colony formation was not observed in resuspended (non-encapsulated) cells under identical culture conditions, indicating that the encapsulation environment supports bacterial survival and localized proliferation over time.

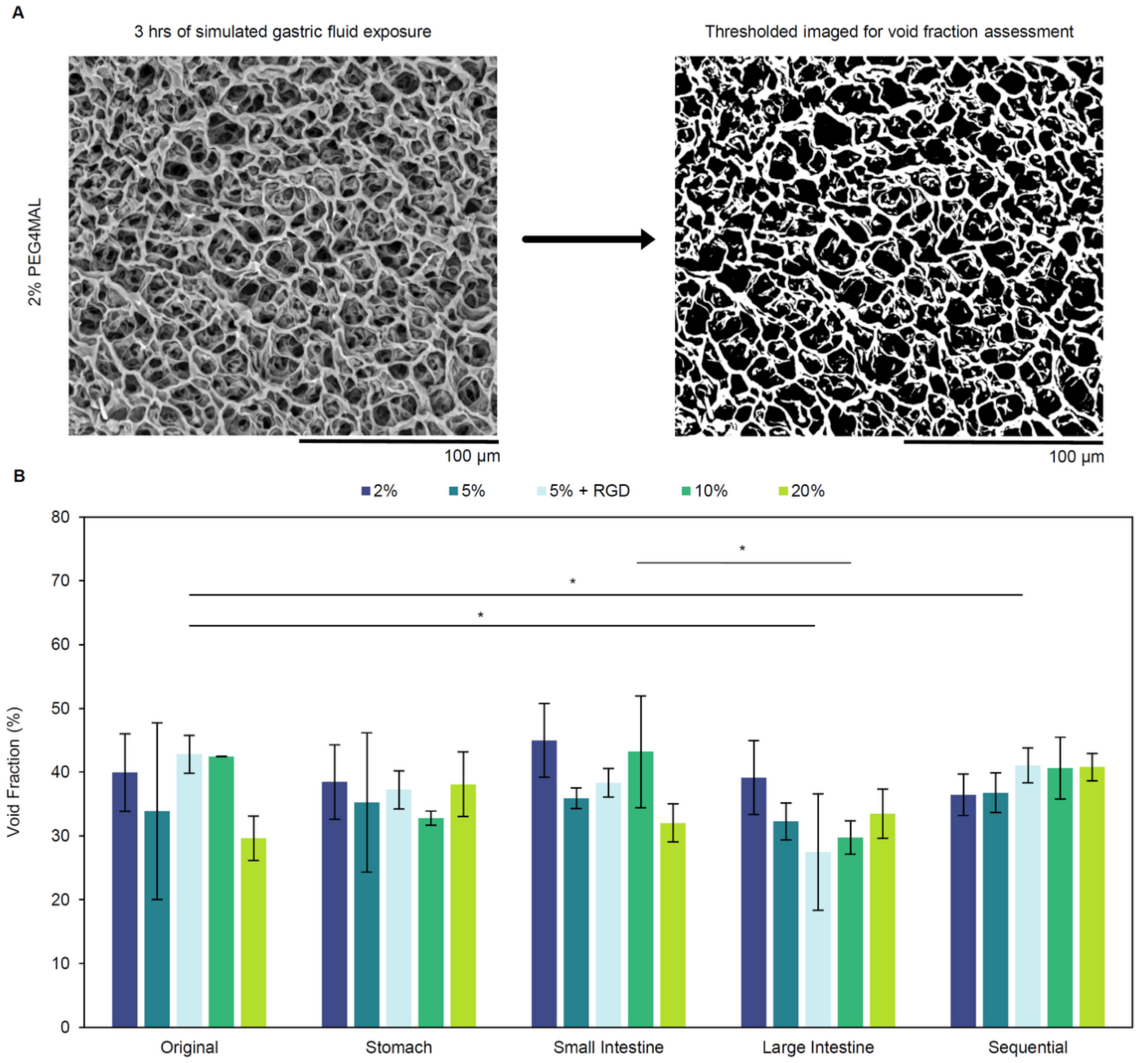

Fig. S1 PEG4MAL degradation post-exposure to simulated gastric and intestinal fluids by A) imaging the surface porosity of PEG4MAL via SEM and; B) the void fraction of the samples following image processing

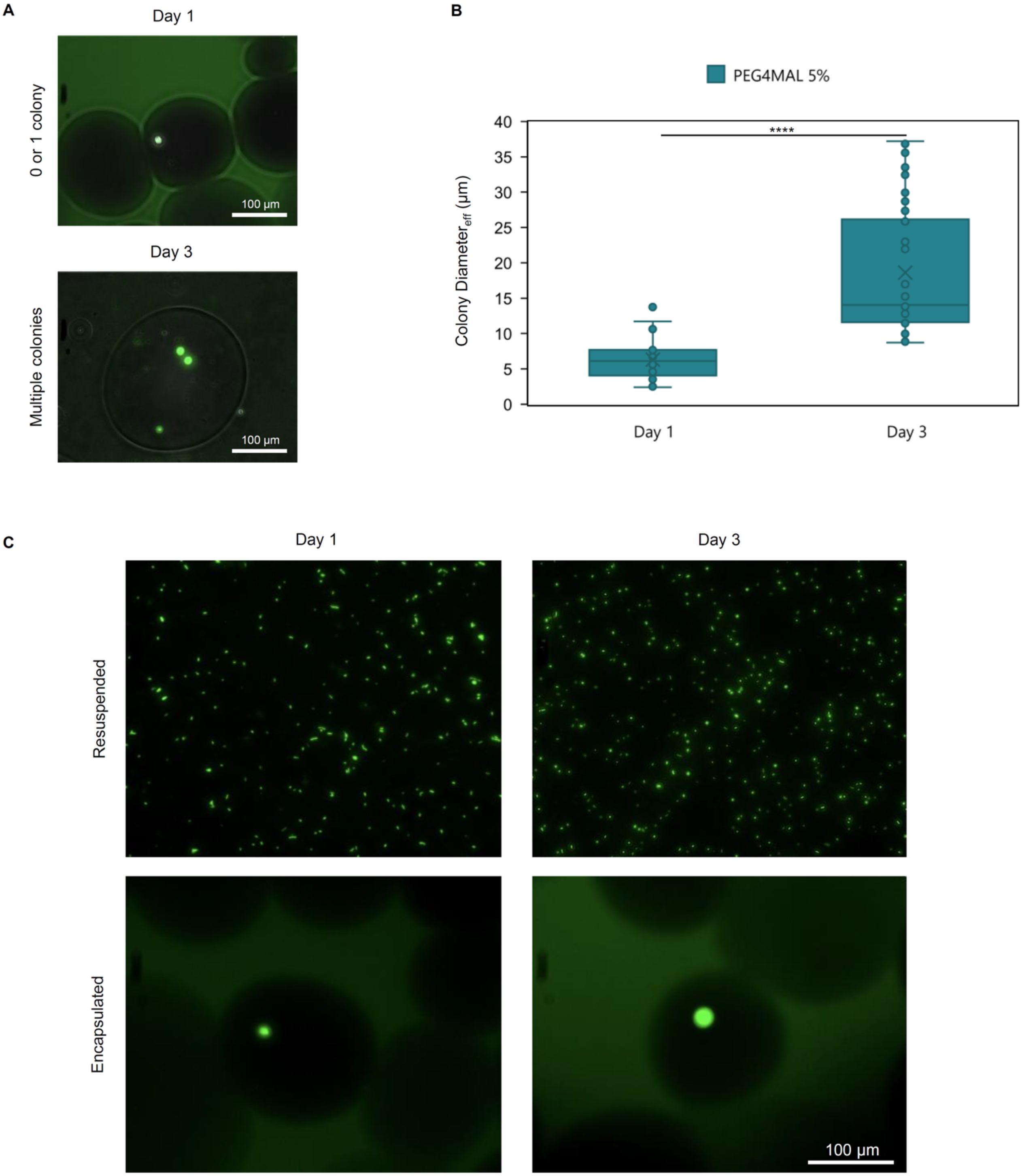

Fig. S2 A) 5% PEG4MAL microbeads containing 0, 1 or more colonies of *C. beijerinckii* at day 1 and 3 after incubation; B) encapsulated *C. beijerinckii* at day 1 and 3 of incubation, $n \geq 30$; C) *C. beijerinckii* aggregation in microbeads vs. resuspended cells

References

Supplementary

[S1] Liu, J., Li, W., Wang, Y., Ding, Y., Lee, A., & Hu, Q. (2021). Biomaterials coating for on-demand bacteria delivery: Selective release, adhesion, and detachment. *Nano Today*, *41*, 101291.

[S2] Fonseca, P. C., & Scherer, G. W. (2015). An image analysis procedure to quantify the air void system of mortar and concrete. *Materials and Structures*, *48*, 3087-3098.
